# Research Protocol: Development, implementation and evaluation of a cognitive behavioural therapy-based intervention programme for the management of anxiety symptoms in South African children with visual impairments

**DOI:** 10.4102/ajod.v4i1.160

**Published:** 2015-07-03

**Authors:** Lisa Visagie, Helene Loxton, Wendy K. Silverman

**Affiliations:** 1 Department of Psychology, Stellenbosch University, South Africa; 2 Yale Child Study Centre, Yale University School of Medicine, United States of America

## Abstract

**Background:**

Childhood anxiety presents a serious mental health problem, and it is one of the most common forms of psychological distress reported by youth worldwide. The prevalence of anxiety symptoms amongst South African youth is reported to be significantly higher than in other parts of the world. These high prevalence rates become even more significant when viewed in terms of children with visual impairments, as it is suggested that children with physical disabilities may be more prone, than their non-disabled peers, for the development of psychological difficulties.

**Objectives:**

The main aim of this study is to develop, implement and evaluate a specifically tailored anxiety intervention programme for use with South African children with visual impairments.

**Method:**

A specifically tailored cognitive-behavioural therapy-based anxiety intervention, for 9–13 year old South African children with visual impairments, will be evaluated in two special schools. The study will employ a randomised wait-list control group design with pre- post- and follow-up intervention measures, with two groups each receiving a 10 session anxiety intervention programme. The main outcome measure relates to the participants’ symptoms of anxiety as indicated on the Revised Child Anxiety and Depression Scale.

**Conclusion:**

If the anxiety intervention programme is found to be effective in reducing symptoms of anxiety, this universal intervention will lay down the foundation upon which future contextually sensitive (South African) anxiety intervention programmes can be built.

## Introduction

A substantial body of literature indicating that childhood anxiety presents a serious mental health problem has now been amassed (Barrett & Turner 2001). Firstly, the literature indicates that anxiety is one of the most common forms of psychological distress reported by youth (Barrett & Sonderegger 2005; Dadds *et al.* 1997). A recent meta analytic prevalence study indicated that an average of 12.3% of school-aged children (ages 6–12) experience significant symptoms of anxiety (e.g., Costello *et al.* 2011, 2003; Heiervang *et al.* 2007; McArdle, Prosser & Kolvin 2004; Mullick & Goodman 2005; Petersen *et al.* 2006). Secondly, although experiencing transient fears and anxieties is deemed to be a normal part of development for most children; for some these experiences may intensify and persist over time, causing interferences in daily functioning (Barrett, Lock & Farrell 2005; Silverman 2011). Thirdly, the experience of childhood anxiety has been associated with a number of somatic difficulties such as fatigue, restlessness, irritability, and sleep disturbances (Beidel, Christ & Long 1991; Pina & Silverman 2004) and psychosocial impairments such as problems with peer relations, low self-esteem, immaturity, impaired academic functioning, and concentration problems (Cooley, Boyd & Gratos 2004; Farrell & Barrett 2007; Messer & Beidel 1994; Motoca, Williams & Silverman 2012). Fourthly, if left untreated, severe symptoms of anxiety can take on a chronic and unremitting course (Barrett & Turner 2004; Cole *et al.* 1998; Weems & Silverman 2013), and many adults who are diagnosed with an anxiety disorder can trace the onset of symptoms back to their childhood (Rapee & Barlow 1993). Given this suggested relationship between childhood anxiety and adult psychopathology, it is essential for symptoms of anxiety to be addressed as early as possible (Cobham 2003), and promising in this regard is the increased evidence pointing to the amenability of childhood anxiety symptoms to brief psychosocial interventions (Lowry-Webster, Barrett & Dadds 2001; Silverman, Pina & Viswesvaran 2008).

Yet, despite these promising results, research indicates that less than 20% of children who require treatment for anxiety will receive clinical intervention (Day & Roberts 1991; Olfsen *et al.* 2003), and of those who do, a large number will terminate prematurely (Kazden 1996; Pina *et al.* 2003), fail to respond (Donovan & Spence 2000; Rey, Marin & Silverman 2011) or, despite treatment, continue to experience recurrent difficulties (Last *et al.* 1996).

In addition, long waiting lists, high costs, high instances of family drop outs, and no show rates also influence access to treatment (Weist 1999). As a result, health policies across the world increasingly promote prevention as the most important direction in which mental health services should move (Barrett & Turner 2004; Lowry-Webster, Barrett & Lock 2003). Prevention offers a positive adjunct to treatment, as prevention programmes can reach a large number of individuals over a shorter period of time; avoid high levels of personal distress for children and their families; and offer a cost-effective and efficient means of intervention prior to the onset of psychopathology (Farrell & Barrett 2007).

The South African prevalence rates of anxiety symptoms are even higher than the above-mentioned international estimates suggest, with Muris and his colleagues (Muris *et al.* 2002) reporting prevalence rates of anxiety symptomology between 22% – 25.6% in children aged 7 to 13 years. These findings were confirmed in subsequent South African studies (Burkhardt, Loxton & Muris 2003; Muris *et al.* 2006). Mostert and Loxton (2008) noted these high prevalence rates and identified the need for a suitable anxiety intervention programme which could be implemented within the South African context. Previous international studies and also the World Health Organization (WHO 2004) had identified Barrett’s (2004) cognitive behavioural therapy (CBT) based FRIENDS Programme to be an efficacious intervention for the reduction of anxiety symptoms in youth (see Briesch, Hagermoser Sanetti & Briesch [2010] for an overview). As a result, Mostert and Loxton (2008) conducted a pilot study to explore the effectiveness and suitability of the FRIENDS Programme for use with South African children. This study reported promising results and its outcomes were in line with the government's white paper on the transformation of the South African health care system, as the study promoted prevention as an important strategy for the enhancement of the mental and physical health of the nation (DOH 1997).

The above-mentioned anxiety factors, their high prevalence rates, and identified need for a suitable intervention programme, become even more significant when viewed in terms of the population of children with visual impairments, who are often marginalised, as previous research involving children with physical disabilities suggests that they are at increased risk of developing psychological difficulties when compared to their non-disabled peers (see Gullone [1996] for an overview). Despite this identified risk, this population has been neglected in previous fear and anxiety research. The last international study to touch on this topic was conducted by Weimer and Kratochwill more than two decades ago (1991) in Wisconsin (USA).

Identifying this gap in the literature, the authors (Visagie *et al.* 2013) conducted an exploratory (baseline) study with South African children with visual impairments. Results indicated that the overall fear profiles of children with visual impairments, and children with normal sight, did not differ significantly (Visagie *et al.* 2013). This similarity indicates that the two populations (children with and children without visual impairments) have similar needs. As the need for a suitable anxiety intervention programme was highlighted in the general South African population (children without visual impairments) (Burkhardt *et al.* 2003; Mostert & Loxton 2008; Muris *et al.* 2006), the same can be said for this specific group. Children with visual impairments also require a suitable and accessible anxiety intervention programme (Loxton, Visagie & Ollendick 2012; Visagie *et al.* 2013).

On closer evaluation of the study by Visagie *et al.* (2013), the need for a suitable anxiety intervention programme was noted to be especially prominent for a specific sub-group of children with visual impairments (i.e. children with severe visual impairment). In their 2012 paper Loxton, Visagie and Ollendick (2012) highlighted significant differences in fearfulness between three groups of visually impaired children who had varying levels of sight (i.e. partially sighted, severely visually impaired and totally blind). These levels of vision can be viewed on a sliding scale (i.e. children who are partially sighted have the most vision; children with severe visual impairment have less vision, but they are not totally blind; and the totally blind children have no level of measureable vision). Children with severe visual impairment (i.e. children who have a degree of light perception and movement detection, but who cannot function optimally without assistance and cannot read print material (WHO 2000), were more fearful than the partially sighted and totally blind children. This group reported the highest number (M = 42.09, SD = 17.84) and also level (M = 171.63, SD = 28.60) of fear. This reported number of fears is almost double the number reported by the children with normal sight (M = 24.66, SD = 13.12). The level of fear in children with normal sight was also lower (M = 146.15, SD = 25.33).

Thus, children with severe visual impairment can be identified as a high-risk sub-group within the broader population of children with visual impairments, for the possible development of later anxiety disorders. A possible suggestion for this sub-group's greater instance of fearfulness could relate to the fact that the visual difficulties, of this group of children, are the most differentiated and difficult to understand. The totally blind children can see nothing; therefore, they need assistance in most unfamiliar situations. On the other hand, partially sighted children usually have enough sight to help themselves and move around independently, and the children with severe visual impairment are in the middle, this group's visual difficulties may be the most complex and most disabling.

Although children with severe visual impairments have a degree of measurable vision, they may find it difficult to function independently in an unfamiliar environment. Therefore, the uncertainty when they are faced with new situations and possibilities may contribute directly to their higher fear reactivity (Bensch 2010; Loxton *et al.* 2012; Sacks *et al.* 2006; Visagie *et al.* 2013). Therefore, it is essential for these children to have access to suitable intervention programmes focused on the development of appropriate coping strategies and problem solving skills to enable them to deal competently with anxiety symptoms when they arise.

Although Mostert and Loxton (2008) obtained favourable outcomes with the FRIENDS Programme with a group of South African children, their study did incur some limitations. It was noted that the FRIENDS acronym (F for feeling worried?, R for Relax and feel good, I for Inner thoughts, E for Explore plans of action, N for Nice work, reward yourself!, D for Do not forget to practise, and lastly S for Stay cool and calm! (Barrett 2004, cited in Mostert 2007), which is aimed to aid children in remembering the coping steps to take when faced with a problem, was difficult for some South African children to remember and problematic to translate into Afrikaans (Mostert & Loxton 2008). Furthermore, Mostert and Loxton (2008) concluded that although the FRIENDS Programme has been used successfully in other countries, South African socio-contextual issues (the high incidence of crime and poverty) should not be ignored – research should in addition be focused on constructing a socially relevant anxiety intervention programme that considers the impact of these factors (Mostert & Loxton 2008). In addition, the FRIENDS Programme is not suitable for use with children with visual impairments, as its content (i.e. cartoons, colouring-in pictures, workbook and visual-based activities) is not very accessible to this specific population.

To summarise, the following six factors motivated the proposed study:

- The high prevalence rates of anxiety symptomology in the general population (especially in South Africa)
- The suggestion that visually impaired children are at increased risk for the development of anxiety disorders when compared to their sighted counterparts
- The lack of anxiety research involving children with visual impairments
- The unique South African socio-cultural context
- The benefits associated with prevention and early intervention with regards to childhood anxiety
- The inaccessibility and appropriateness of existing anxiety intervention programmes.

As a result, the proposed study will aim to develop, implement and evaluate an anxiety prevention or early intervention programme (from here on referred to as an anxiety intervention programme), which is specifically tailored to meet the needs of children with visual impairments within the South African context. As far as the researchers can ascertain, no studies focussed on anxiety prevention and early intervention programmes, for children with visual impairments, have been conducted previously.

Thus, the primary aim of the study is to develop, implement and evaluate a specifically tailored anxiety intervention programme for use with South African children with visual impairments. This aim will unfold in the following three steps:

1. develop an accessible anxiety intervention programme which can be used with South African children with visual impairments (programme development phase).
2. implement this anxiety intervention programme successfully (programme implementation phase).
3. evaluate the effectiveness of this anxiety intervention programme in maintaining emotional health (i.e. preventing an increase in symptoms of anxiety as measured on the Revised Child Anxiety and Depression Scale [RCADS] [Chorpita *et al.* 2000] [programme evaluation phase]).

Specifically, it is expected that firstly, there will be a significant reduction in anxiety scores on the RCADS from Time 1 (T1) to Time 2 (T2) for the immediate intervention group (IIG), and secondly, there will be a significant reduction in anxiety scores on the RCADS from T2 to T3 for the delayed intervention group (DIG).

## Method

A specifically tailored CBT-based anxiety intervention for South African children with visual impairments will be evaluated in two special schools that cater for children with visual impairments. This study is set up as a randomised wait-list control group design with pre-, post-and follow-up intervention measures, with 60 participants randomly assigned to an IIG (*n* = 30) and a DIG (*n* = 30). Before implementation of the programme 10 participants from each group will be randomly selected to take part in a focus group. The focus group will be aimed at obtaining the participants’ personal inputs and to pilot certain sections of the programme before final implementation. Both of the programme's groups will receive the 10 session anxiety intervention programme**.** The participants will complete baseline (T1), post intervention (T2 for IIG and T3 for DIG) and 3 month (T3 for IIG and T4 for DIG) or 6 month (T4 only for IIG) follow-up assessments.

The research design can be represented as follows:

R  IIG T1   X  IIG T2     IIG T3  IIG T4

R  DIG T1     DIG T2  X  DIG T3   DIG T4

**R = Randomisation**

**X = intervention**

### Participants

It is envisaged that the final sample will consist of approximately 60 children; 30 children in the IIG and 30 children in the DIG who attend Grades 4–7 (aged 9–13 years) at two schools (School 1 and School 2), for children with visual impairments in the Western Cape Province of South Africa. Letters explaining the study will be sent to the two identified schools, and the necessary written consent for participation will be requested from the school principals and school governing bodies.

Parents or guardians of the identified participants (children attending Grades 4–7) will be provided with an information letter explaining the study and a consent form giving their child permission to take part. Identified participants will also be provided with information about the study and they will be required to give written assent before they are delivered the programme. Thus, dual parent-child consent and assent will be required for inclusion. This study received approval (HS888/2013) from the Research Ethics Committee: Human Research (Humaniora) at the Stellenbosch University (Institutional Review Board Number: IRB0005239), and the Western Cape Education Department in South Africa (Reference: 20130507–10635).

### Inclusion criteria

The criterion for inclusion in the study will be one of universal prevention where children will be included in the programme regardless of their anxiety status. Universal prevention is a positive approach to social-emotional learning as it aims to build strengths in all children through the enhancement of known skills and protective factors (Farrell & Barrett 2007). However, a distinction about the programme description can be made. Based on each participants T-score on the RCADS (Chorpita *et al.* 2000) at T1, the programme can be described as either an early intervention (T-score above 65) or prevention programme (absence of significant symptoms of anxiety). Furthermore, participants should be aged between 9 and 13 (attend Grades 4–7), and be able to read and write. The reason for this requirement is that the measuring instruments are self-report surveys and participants must read the questions and complete these themselves. Lastly, with the exception of visual impairment, participants included in the study should have no other disability.

### Randomisation

Randomisation will be applied within schools with an immediate intervention and delayed intervention group within each school. Participants will be assigned to either the IIG or the DIG at their respective schools. The IIGs from School 1 and School 2 will be combined to form one large IIG, and the DIGs from School 1 and School 2 will be combined to form one large DIG.

## Programme intervention

A specifically tailored, group-based, anxiety intervention programme for children with visual impairments will be implemented as a universal intervention. The anxiety intervention programme, which is based on a CBT model, was developed over a period of six months. CBT-based interventions employ both cognitive (e.g. positive self-talk) and behavioural (e.g. relaxation) techniques to modify cognitions, behaviour and affect (Kendall 1993; Nevid, Rathus & Green 2000; Silverman & Kurtines 1996). Thus, CBT-based interventions aim to teach children to identify maladaptive thoughts and to replace these thoughts with more positive ones (Mash & Wolf 2005). In addition, one of the greatest benefits of CBT is its longevity, as CBT is focussed on equipping people with a set of coping skills that can be used both now and in the future. Furthermore, CBT-based interventions are usually shorter-term interventions, making them more cost-effective and appealing (Farrell & Barrett 2007; Ollendick & King 1998; Silverman *et al.* 2008). For these reasons, CBT was the intervention modality of choice for the study.

The eight key concepts of CBT as described by Barrett and Turner (2001) are central to the anxiety intervention programme's theoretical base, and include:

1. recognition of the link between thoughts and feelings
2. identifying feelings
3. relaxation strategies
4. cognitive restructuring
5. attention training
6. problem-solving
7. self-reward
8. relapse prevention.

The programme's core theme relates to the enhancement and development of skills or competencies which can be employed when confronted with difficult situations, whether they relate to fears or worries, daily hassles (e.g. struggling with a difficult homework assignment), or stressful and aversive life events (e.g. moving to a different school or facing family conflict) (Barrett & Turner 2001). In addition to incorporating the eight key concepts of CBT, the anxiety intervention programme lent and built on ideas and concepts adapted from already existing CBT-based approaches and programmes, such as:

- Kendall's Coping Cat Programme (Kendall 1990)
- Barrett's Coping Koala Programme (Barrett 1995)
- Silverman and Kurtine's Transfer of control approach (Silverman & Kurtines 1996)
- Stallard's Think Good-Feel Good Programme (Stallard 2002)
- Barrett's FRIENDS For Life Programme (Barrett 2004)
- Rapee's Cool Kids^®^ Programme (Rapee *et al.* 2006).

The challenge in this adaptation was not only to translate the eight key concepts of CBT into a child-friendly and age-appropriate programme, aimed at combating anxiety symptoms, but also to present the programme in a format that is accessible and appealing to children with visual impairments. As an alternative to reading and colouring in pictures (which is the norm in most of the programmes to date) the adapted programme makes use of clay (and other tactile media), toys, role-plays, games, songs, narratives and other creative techniques as alternative forms of engagement. As the FRIENDS acronym is not suitable for use in the proposed study the title for the adapted intervention programme is the P& M programme (PAM = Positive & Motivating, and in Afrikaans Positief & Motiverend). Activities in the P& M programme are aimed at teaching children with visual impairments practical skills, such as: identifying feelings; relaxation skills; identifying unhelpful and helpful thoughts; and how to deal with daily problems and challenges (Stallard *et al.* 2007).

### Immediate intervention group (IIG)

All assenting children in the IIG will receive a 10–session CBT-based, group programme, each session will last approximately 60 minutes. The sessions will take place twice a week for five weeks. Groups consist of 8–10 participants. The programme will be delivered at schools during a time negotiated with the school. The Programme will start with activities that promote group cohesion and teamwork (session 1). In sessions 2 and 3 psycho-education, regarding emotions and thoughts, is given, and in session 4 bodily reactions to anxiety are addressed and relaxation exercises are introduced. Group participants are encouraged to practice these relaxation exercises throughout the duration of the programme. The next step (session 5) relates to recognising inner thoughts and the promotion of positive thinking. Session 6 introduces the concept of cognitive restructuring and introduces participants to problem solving skills. Self-evaluation and reinforcement is introduced in session 7. Sessions 8 and 9 allow for group participants to repeat and practice the new skills they have learnt, and session 10 covers relapse prevention and there is a party to celebrate the completion of the programme. At the start of the programme, parents or guardians and teachers will also be invited to attend an additional psycho-educational information session. This information session will provide an overview of the anxiety intervention programme, discuss the rationale underlying CBT-based programmes and explain the skills that children will be taught.

### Delayed intervention group (DIG)

Participants in the DIG will be delivered the same intervention programme straight after the programme has been delivered to participants in the immediate intervention group, and they will also be asked to complete three questionnaires on four occasions, together with the children in the IIG.

### Group leader (Trainer)

The programme will be delivered by the first author and a research facilitator (a post-graduate student in psychology) in either English or Afrikaans, depending on the children's language of schooling. The first author is a registered counselling psychologist with ample experience and knowledge relating to developmental psychology and CBT. The group leader will be blind to all assessment measures. The reason the first author herself will conduct the intervention is that there are no other qualified psychologists available who are visually impaired, and who are attuned to the social and cultural context of the participants.

## Programme evaluation

As mentioned above, all assenting children from School 1 and School 2 will randomly be assigned to 1 of 2 groups at their respective school (an IIG or a DIG). The IIG groups from School 1 and School 2 will be combined to form one large IIG, whilst the DIG groups from School 1 and School 2 will be combined to form one large DIG.

The evaluation phase will comprise both a quantitative and qualitative component (more information about the qualitative component is given below). For the quantitative evaluation, all assenting children in the IIG and DIG (approximately 60 participants) will be administered a short biographical questionnaire (only at T1) and three self-report questionnaires at pre (T1), post (T2), and follow-up (T3 and T4) intervention. Children in the IIG will receive the intervention programme before the children in the DIG. It is envisaged that the programme will be delivered to the two groups (the IIG and DIG) of children according to the following outline:

- Initial assessment of all children, IIG and DIG (T1)
- Children in the IIG receive the anxiety intervention programme (5 weeks)
- Re-assessment of all children, IIG and DIG (T2)
- Children in the DIG receive the anxiety intervention programme (5 weeks)
- Re-assessment of all children, IIG and DIG (T3)
- Final assessment of all children, IIG and DIG (T4 – It will be 6 months after the children in the IIG received the intervention and 3 months after the children in the DIG received the intervention). Resulting from time constraints, where all assessments should be conducted within one academic year, there will be no 6 month follow-up for the DIG, and no wait period for the IIG.

It will be possible to measure:

- The stability of the measures when untreated over time (The DIG will act as a wait-list control group using T1 and T2 comparisons)
- The impact of the anxiety intervention programme immediately after completion (using immediate pre and post-data from both groups – IIG [T1 and T2]; and DIG [T2 and T3])
- The maintenance of the effects of the anxiety intervention programme over time following intervention (IIG using T2, T3 [3 month] and T4 [6 month] comparisons, and DIG using T3 and T4 [3 month] comparisons)

The following six hypotheses are to be tested during the evaluation phase of the proposed study (four hypotheses pertain to between group effects and two hypotheses pertain to within group effects).

### Between group effects

- Firstly, it is hypothesised that there will be no significant differences between the scores obtained by the IIG and DIG on the RCADS at T1.
- Secondly, anxiety scores obtained by the IIG on the RCADS (post intervention) will be significantly lower than the anxiety scores obtained by the DIG on the RCADS at T2.
- Thirdly, there will be no significant differences in the anxiety scores of the IIG at T2 and DIG on the RCADS at T3.
- Fourthly, results obtained on the RCADS at post-intervention (T3) will be retained at the three (DIG) and six month (IIG) follow-ups (T4).

### Within group effects

- Within the IIG there will be a significant reduction in anxiety scores on the RCADS from T1 to T2.
- Within the DIG there will be a significant reduction in anxiety scores on the RCADS from T2 to T3.

For the qualitative process evaluation of the programme, all ten intervention sessions will be video recorded, and the verbal content of the recordings will be transcribed verbatim. The transcriptions will be analysed to identify recurring themes (thematic content analyses). After completion of the ten sessions participants will also be asked to complete a series of short questions compiled by the researcher. In addition, the researcher and the research assistant (observer) will also keep a detailed record (in the form of process notes) of all sessions. The intervention programme will be completely manualised and a quantifiable measure, to measure adherence to the treatment protocol, will be developed and implemented. These qualitative components will be included in the research to ensure fidelity and to provide as many pointers as possible to aid in the understanding of an under-researched problem, namely anxiety experienced by children with visual impairments.

Hartley and Muhit (2003) state that the inclusion of a qualitative component in research can help bridge the gap between scientific study and clinical practice, and may assist in gaining a better understanding of the phenomena under investigation. They further argue that qualitative methodology is needed to collect cultural and disability specific data which may not be easily obtained through quantitative methods, as the low prevalence rates of some disabilities make it very difficult to draw statistically significant conclusions from quantitative data (Philander 2007). Hartley and Muhit (2003:103) further emphasise that qualitative sources of data provide an opportunity not only to listen, but also to include the voices of vulnerable populations in programme planning. They stated this as follows: ‘it [*qualitative data*] educates quantitative researchers about the people and their perceptions, beliefs and practices’.

## Data collection

Questionnaires will be made available in four different input modes relating to the individual participant's specific needs and degree of visual impairment. In some cases the original print versions of the questionnaires will be provided and read by means of a magnifying aid, questionnaires will also be put in a large print format (A3), and for those participants who cannot read print, the questionnaires will be made available in braille or in an audio format. The modes of response will also be adapted to suit the individual participant's communication needs. Participants who are able to read the standard or large print copies of the questionnaires can indicate their answers in the spaces provided, and for the participants using braille or recorded versions of the questionnaires, specially prepared answer sheets will be made available.

In previous studies (King, Gullone & Stafford 1990; Matson *et al.* 1986; Ollendick, Matson & Helsel 1985; Wilhelm 1989) where the fears and anxieties of visually impaired children were assessed using self-report measures (e.g. The Revised Fear Survey Schedule for Children, FSSC-R; The South African Fear Survey Schedule for Children, FSSC-SA; and the Revised Children's Manifest Anxiety Scale, RCMAS) similar adaptations were made with great success. Children will complete the questionnaires at their school with the help of the research facilitators. Guidelines put forth by Visagie and Loxton (2014) about the child-friendly assessment and the accomodations needed for children with visual impairments to complete self-report surveys, will be implemented and followed throughout the process of data collection.

The questionnaires will be administered to all participants (IIG and DIG) on four occasions:

- Immediately before delivering the anxiety intervention programme to the IIG (T1) (Baseline)
- Immediately after delivering the anxiety intervention programme to the IIG, and before delivering the anxiety intervention programme to the DIG (T2)
- Immediately after delivering the anxiety intervention programme to the DIG (T3)
- At follow-up, six months after delivering the programme to the IIG, and three months after delivering the programme to the DIG (T4)

### Participant outcomes

The primary outcome measure in the study relates to changes in levels of symptoms of anxiety and depression on the Revised Child Anxiety and Depression Scale (RCADS) (Chorpita *et al.* 2000). The 30 items of the RCADS assess symptoms across six domains of anxiety and depression in children (aged 6–18), including:

- social phobia
- separation anxiety
- obsessive compulsive disorder
- panic disorder
- generalised anxiety disorder
- major depressive disorder.

Items are rated on a four-point Likert scale. Psychometric properties of the RCADS are good; the six RCADS scales were found to have adequate internal consistency (with all alphas in the .70 and .80 range) and test-retest stability (with one-week test-retest correlation coefficients ranging from .65 to .80) (Chorpita *et al.* 2000; Muris, Meesters & Schouten 2002). The RCADS is freely available on the Internet for download, and permission to translate the questionnaire into Afrikaans was obtained from the authors. Translations were written by a registered clinical psychologist who is fluent in both English and Afrikaans in accordance with the Brislin (1980) back-translation method.

Secondary outcome measures relate to children's worry and self-efficacy measured by the Penn State Worry Questionnaire for Children (PSWQ-C) (Chorpita *et al.* 1997) and Self-Efficacy Questionnaire for Children (SEQ-C) (Muris 2001) respectively. The PSWQ-C (Chorpita *et al.* 1997) is an 11–item questionnaire which assesses the tendency of children (aged 7–17) to engage in excessive generalised and uncontrolled worry. Respondents are asked how often each item applies to them by indicating answer options on a four-point Likert scale. Chorpita *et al.* (1997) found the PSWQ-C to have favourable psychometric properties, reporting good internal consistency (*A* = .88) and test-retest reliability (*R* = .92) (over a one-week interval). The PSWQ-C is freely available on the Internet for download, and permission was obtained from the authors to translate the questionnaire into Afrikaans. Once again translations were written in accordance with the Brislin (1980) back-translation method. Muris’s (2001) SEQ-C is a 24 item self-report scale. The SEQ-C measures children's self-efficacy across three domains including: social, academic and emotional self-efficacy. Respondents are required to rate each of the 24 items on a five-point Likert scale. The reliability of the scale ranges from .88 for total self-efficacy and between .85 and .88 for the three sub-scales (Muris 2001). The SEQ-C has been translated into Afrikaans and has been used successfully in previous studies (Le Roux 2013; Muris 2001).

### Teacher outcomes

An additional outcome relates to emotional and behavioural difficulties experienced by group participants. The Strengths and Difficulties Questionnaire (SDQ) is a widely used, brief behavioural screening questionnaire aimed at detecting behavioural and emotional difficulties in youth (aged 3–16 years) (Mostert 2007). The SDQ consists of 25 items which can be divided into five sub-scales of five items each. Four sub-scales comprise the most important domains of child psychopathology, including:

- emotional symptoms
- conduct problems
- hyperactivity and inattention
- peer relationship problems.

Scores on these sub-scales add up to a total difficulties score. The remaining subscale (pro-social behaviour) measures the child's strengths in social interactions (Goodman 1997). Items are scored on a three-point Likert scale (Goodman *et al.* 2003). Class teachers will be asked to complete the informant version of the SDQ (Mostert 2007) on two occasions for each participant, before and directly after implementation of the anxiety intervention programme. The informant version of the SDQ assesses the teacher's perception of whether a child has a problem or not. Focus is given to chronicity, distress, social impairment and burden. The SDQ has been found to have favourable psychometric properties and the questionnaire was deemed to have good construct validity, as it was found to have substantial correlations with other indices of psychopathology (Goodman, Meltzer & Bailey 1998; Goodman, Renfrew & Mullick 2000; Goodman *et al.* 2003).

### Qualitative outcomes

The final outcome measure relates to the qualitative evaluation of the programme. It is not only important to determine whether a programme works, but also whether the participants consider the programme to be beneficial and worthwhile (Barrett & Turner 2001). For this reason, after completion of the 10 sessions, oral instructions will be given to participants to complete a series of short questions compiled by the researcher. These questions provide an opportunity to obtain qualitative feedback from the participants relating to their experience of, and satisfaction with, the anxiety intervention programme.

### Statistical analyses

The Statistical Package for the Social Sciences (SPSS) (Van Leeuwen *et al.* 2006) will be used to calculate descriptive and non-parametric statistics. As a result of the envisaged small sample size (*N* = 60), a Mann-Whitney test will be used to determine the significance of differences between the quantitative pre-and-post intervention measures (George & Mallery 2006). Pre-intervention (T1) scores will be analysed to determine the equivalence of the IIG and the DIG, before the intervention and change scores (difference between the pre-and post-intervention scores) for the IIG and DIG will be compared to evaluate change as a result of the intervention programme. Qualitative data will be analysed by means of thematic content analyses.

## Discussion

This study will employ a randomised wait-list control group design with pre and post-intervention measures to evaluate the effectiveness of a specifically tailored, group-based, CBT anxiety intervention programme for 9–13 year old South African children with visual impairments. It is hypothesised that children in the IIG will experience significantly reduced symptoms of anxiety when compared to children in the DIG.

### Strengths and limitations

The major strength of this study is that it is the first of its kind, with no studies focussed on anxiety prevention and early intervention programmes for children with visual impairments having been conducted previously. Thus, this research has a novel methodology and it will fill an important gap in the literature by developing and promoting critically needed, specifically tailored anxiety prevention strategies which can be used by children with visual impairments. Expected advantages for participants include:

- a reduction in anxiety symptomology
- an increase in self-efficacy
- the acquisition of helpful problem solving skills and coping strategies.

Thus, the programme is aimed at optimising human development and, in this way, contributing to future wellbeing and potentially reducing the prevalence of anxiety in the population of children with visual impairments. Rather unique to the study's design is the qualitative feedback from the participants and facilitators relating to their experience of and satisfaction with the anxiety intervention programme. These qualitative data will provide much-needed information to aid in the understanding of an under-researched problem, namely anxiety experienced by children with visual impairments.

One limitation is the study's small sample size. This is the result of logistical and geographical constraints. South Africa is a large country and schools for children with visual impairments are widely dispersed which, thus, limits the author's capacity to travel to numerous schools on a week-by-week basis.

### Implications for practice

If it is found that this anxiety intervention programme is effective in reducing symptoms of anxiety in South African children with visual impairments, this group-based universal intervention will lay down the foundation upon which future contextually sensitive (South African) anxiety intervention programmes can be built. Furthermore, a recent article by Lyner-Cleophas *et al.* (2014) highlighted the importance of developing coping skills at an early age for academic success later in life.

## Conclusion

This study will evaluate the effectiveness of a specifically tailored, group-based, CBT anxiety intervention programme for South African children with visual impairments, and the results rendered by the study will provide insights into the effectiveness of the anxiety intervention, and determine its suitability for future use within the South African context.
